# Plasma amyloid assay as a pre-screening tool for amyloid positron emission tomography imaging in early stage Alzheimer’s disease

**DOI:** 10.1186/s13195-019-0566-0

**Published:** 2019-12-27

**Authors:** Szu-Ying Lin, Kun-Ju Lin, Po-Chen Lin, Chin-Chang Huang, Chiung-Chih Chang, Yi-Chung Lee, Ing-Tsung Hsiao, Tzu-Chen Yen, Wen-Sheng Huang, Bang-Hung Yang, Pei-Ning Wang

**Affiliations:** 1 Department of Neurology, Taipei Municipal Gan-Dau Hospital, Taipei, Taiwan; 2 Department of Nuclear Medicine and Molecular Imaging Center, Linkou Chang Gung Memorial Hospital, Tao-Yuan, Taiwan; 3 Healthy Aging Research Center and Department of Medical Imaging and Radiological Sciences, College of Medicine, Chang Gung University, Tao-Yuan, Taiwan; 4 Department of Neurology, Neurological Institute, Taipei Veterans General Hospital, Taipei, Taiwan; 5 Department of Neurology, Linkou Chang Gung Memorial Hospital and University, Tao-Yuan, Taiwan; 6 Department of Neurology, Kaohsiung Chang Gung Memorial Hospital, Kaohsiung, Taiwan; 7 Department of Neurology, School of Medicine, National Yang-Ming University, Taipei, Taiwan; 8 Brain Research Center, National Yang-Ming University, Taipei, Taiwan; 9 Department of Nuclear Medicine, Taipei Veterans General Hospital, Taipei, Taiwan; 10 Department of Biomedical Imaging and Radiological Sciences, National Yang-Ming University, Taipei, Taiwan; 11 Aging and Health Research Center, National Yang-Ming University, Taipei, Taiwan; 12 Division of General Neurology, Department of Neurological Institute, Taipei Veterans General Hospital, Taipei, Taiwan

**Keywords:** Amyloid PET, *APOE*, Plasma Aβ, Biomarkers, MCI, AD

## Abstract

**Introduction:**

Due to the high cost and high failure rate of ascertaining amyloid positron emission tomography positivity (PET+) in patients with earlier stage Alzheimer’s disease (AD), an effective pre-screening tool for amyloid PET scans is needed.

**Methods:**

Patients with mild cognitive impairment (*n* = 33, 24.2% PET+, 42% females, age 74.4 ± 7.5, MMSE 26.8 ± 1.9) and mild dementia (*n* = 19, 63.6% PET+, 36.3% females, age 73.0 ± 9.3, MMSE 22.6 ± 2.0) were recruited. Amyloid PET imaging, Apolipoprotein E (*APOE*) genotyping, and plasma amyloid β (Aβ)_1–40_, Aβ_1–42_, and total tau protein quantification by immunomagnetic reduction (IMR) method were performed. Receiver operating characteristics (ROC) analysis and Youden’s index were performed to identify possible cut-off points, clinical sensitivities/specificities, and areas under the curve (AUCs).

**Results:**

Amyloid PET+ participants had lower plasma Aβ_1–42_ levels than amyloid PET-negative (PET−) subjects. *APOE* ε4 carriers had higher plasma Aβ_1–42_ than non-carriers. We developed an algorithm involving the combination of plasma Aβ_1–42_ and *APOE* genotyping. The success rate for detecting amyloid PET+ patients effectively increased from 42.3 to 70.4% among clinically suspected MCI and mild dementia patients.

**Conclusions:**

Our results demonstrate the possibility of utilizing *APOE* genotypes in combination with plasma Aβ_1–42_ levels as a pre-screening tool for predicting the positivity of amyloid PET findings in early stage dementia patients.

## Background

Beta-amyloid (Aβ) plaque deposition in the brain is the pathological hallmark of Alzheimer’s disease (AD) [1–3]. Disease-modifying drugs with anti-amyloid effects are designed to eliminate aggregated Aβ [4–6]. The results of phase I and phase II trials of these anti-amyloid drugs reveal their ability to eliminate Aβ plaques in the brains of patients with prodromal AD [7–9]. To prove the efficacy of these drugs, trials target prodromal AD patients with confirmed Aβ pathology for recruitment. Comprehensive neuropsychological assessment identified participants’ characters and severities [10, 11]. Aβ pathology is confirmed by amyloid positron emission tomography (PET) [12–14]. However, amyloid PET scans are expensive, and the availability of amyloid PET is limited. Widespread use of amyloid PET imaging in the pre-screening phase of clinical trials is thus not feasible. A pre-screening tool with low cost and high efficiency for evaluating the probability of PET positivity/negativity (PET+/PET−) is thus needed.

Cerebrospinal fluid (CSF) biomarkers are an alternative way to diagnose cerebral Aβ pathology. Many studies showed that the Aβ_1–42_ concentration in the CSF decreases in patients with dementia due to AD [14–17]. The accuracy of discriminating AD from healthy subjects by the CSF Aβ_1–42_/Aβ_1–40_ ratio can reach 80% [14]. Furthermore, the negative correlation between the CSF Aβ_1–42_/Aβ_1–40_ ratio and Aβ deposition assessed by amyloid PET is strong [18–20]. However, lumbar puncture is an invasive procedure that may cause discomfort and side effects such as headache, back pain, swelling, and bruising. Therefore, CSF biomarkers are not widely assessed in clinical practice. Alternative methods of pre-screening that are comfortable, low risk, and low cost are needed; these pre-screens can be administered before high-cost amyloid PET scans, especially in trials for amyloid modulation therapy.

Blood tests are easy, low cost, low risk, and highly available. A total of 30–50% of blood Aβ protein may come from the brain [21]. However, blood Aβ_1–42_ and Aβ_1–40_ levels are extremely low, and it is difficult to determine blood Aβ levels precisely [22–28]. Some reports have indicated that immunomagnetic reduction (IMR) is sensitive enough to assay ultra-low concentration of Aβ_1–42_ and Aβ_1–40_ in human plasma [23–27]. Using this technique, the concentrations of plasma Aβ_1–42_ have been shown to differentiate healthy elderly subjects from subjects with all stages (mild, moderate, and severe) of AD [25]. A previous study demonstrated the accuracy of using the plasma Aβ_1–42_/Aβ_1–40_ ratio as a diagnostic parameter in differentiating healthy subjects from patients with either mild or severe AD [25]. In addition, the plasma Aβ_1–42_/Aβ_1–40_ ratio increases with increasing amyloid load, as assessed by amyloid PET imaging in normal subjects and patients with dementia due to AD [28]. These results show the feasibility of assaying plasma Aβ_1–42_ and Aβ_1–40_ for evaluating whether to perform amyloid PET scans.

Genetic factors influence amyloid aggregation in both normal subjects and patients with AD. It has been reported that the Apolipoprotein E (*APOE*) ε4 allele is associated with greater Aβ deposition in the brain [29–31]. Once Apolipoprotein E (*APOE*) protein binds with Aβ, the complex becomes unstable and easily forms fibrillary Aβ [32–34]. The co-existence of *APOE* and Aβ in amyloid plaques is supported by histopathological findings [35]. Previous studies have found subjects with the *APOE* ε4 allele have a higher chance of presenting with amyloid PET+ than those without ε4 [14, 36].

In the present study, we sought to develop an algorithm using plasma Aβ_1–42_, Aβ_1–40_, tau, and *APOE* genotypes as a pre-screening tool to enhance the accuracy of predicting amyloid PET+ in clinically suspected mild cognitive impairment (MCI) and mild AD patients.

## Methods

### Recruitment of subjects

Through the Alzheimer’s Disease Neuroimaging Initiative in Taiwan (T-ADNI), subjects were enrolled at Taipei Veterans General Hospital (Taipei VGH), Linkou Chang Gung Memorial Hospital (CGMH), and Kaohsiung CGMH.

Enrolled subjects were required to be 55 to 90 (inclusive) years of age and to have at least 6 years of education. All subjects were interviewed by neurologists to obtain an extensive clinical history. Demographics, family history, physical examination, neurologic examination, Hachinski ischemic score, vital signs, and blood for screening labs (hematology, chemistry panel, vitamin B_12_, syphilis rapid plasma reagin, thyroid-stimulating hormone, and free thyroxine) were collected. A standard neuropsychological evaluation was performed. The screening laboratory and magnetic resonance (MR) imaging examinations were used to rule out other major neuropathologies such as tumors, strokes, severe white matter disease, and inflammation, but they were not used to diagnose dementia. All subjects were required to have no history of major brain trauma, brain tumor, stroke, epilepsy, alcoholism, major psychiatric illness, or other systemic diseases that affect cognitive function.

Diagnostic criteria for amnestic mild cognitive impairment (aMCI) and mild dementia were in accordance with the criteria used in Alzheimer’s Disease Neuroimaging Initiative (ADNI). Subjects underwent a series of screening evaluations including the Geriatric Depression Scale, a Mini-Mental State Examination (MMSE), the Chinese version of the Wechsler Memory Scale-III (WMS-III), and the immediate and delayed conditions of the Logical Memory (LM) task. A Clinical Dementia Rating Scale (CDR) score was obtained. All dementia patients and the majority of amnestic MCI patients fulfilled the National Institute on Aging and the Alzheimer’s Association (NIA-AA) recommended criteria for dementia due to AD and for MCI, respectively [10].

Only subjects with CDR scores of 0.5 and MMSE scores of 20–30 were analyzed in this study. Thus, all subjects in this study were clinically suspected MCI or mild AD patients. The demographic information for these early stage AD patients is listed in Table 1. Subjects were divided into two groups according to amyloid PET results.

**Table 1:** Demographic information for enrolled clinically suspected early stage AD subjects

Amyloid PET	Positive	Negative	All
Numbers (% female)	22 (50.0%)	30 (36.7%)	52 (42.3%)
Age (years)	72.1 ± 7.6	71.9 ± 9.7	72.0 ± 8.8
Education (years)	12.0 ± 4.3	11.0 ± 3.6	11.4 ± 3.9
Clinical stage			
aMCI	8	25	33
Mild dementia	14	5	19
*APOE* ε4 carrier	12	2	14
CDR	0.5	0.5	0.5
MMSE	24.0 ± 2.7	27.0 ± 2.2^†^	25.8 ± 2.8
Logical memory delayed recall	5.41 ± 3.92	8.40 ± 5.12*	7.13 ± 4.84

*Abbreviations*: *AD* Alzheimer’s disease, *CDR* Clinical Dementia Rating Scale, *MMSE* Mini-Mental State Examination, *PET* positron emission tomography

*P value < 0.05

^†^*P* value < 0.001

### Image data acquisition

The radiosynthesis of ^18^F-florbetapir [37] and amyloid PET data acquisition [38] were described previously by our group. All PET images were acquired from a single site, and the scanner was calibrated with a Hoffman brain phantom. The ^18^F-florbetapir PET scan comprised a 10-min acquisition period (acquired in 2 × 5 min frames) beginning 50 min following 10 mCi injection of the ^18^F-florbetapir tracer. Imaging was performed on a Biograph mCT PET/CT scanner (GE Healthcare, Milwaukee, USA). Each PET image was obtained using the three-dimensional ordered subset expectation maximization (3-D OSEM) reconstruction algorithm (four iterations, 24 subsets; Gaussian filter 2 mm; zoom: three) with CT-based attenuation correction, as well as scatter and random corrections, with a matrix size of 400 × 400 × 148 and a voxel size of 0.68 × 0.68 × 1.5 mm^3^. Structural MRI scans were acquired using a uniform scanning protocol that minimized and accounted for between-site differences in MRI systems. T1-weighted MRI images were obtained for all subjects to obtain useful anatomical information and enable coregistration with PET images.

### Amyloid PET image processing

All PET image data were processed and analyzed using PMOD image analysis software (version 3.7, PMOD Technologies Ltd., Zurich, Switzerland), including MR-based spatial normalization to the Montreal Neurological Institute (MNI) MRI template [39]. Seven volumes of interest (VOIs), the frontal, anterior cingulate, posterior cingulate, precuneus, parietal, occipital, and temporal areas, were selected, and the regional standardized uptake value ratio (SUVR) using the whole cerebellum as the reference region was calculated for each VOI. Moreover, the average SUVR from these seven cerebral cortical VOIs was computed to yield an estimate of global cortical SUVR for further analysis.

The PET images were interpreted by an experienced, blinded nuclear medicine physician (Kun-Ju Lin). A 5-point visual scale was used to classify the amyloid loading, from 0, indicated no tracer retention in cortical gray matter, to 4, indicated high levels of cortical amyloid accumulation. Visual rating scores of 2–4 were considered indicative of amyloid PET+ brains, and ratings of 0–1 were considered negative for amyloid PET [37].

### Preparation of human plasma

Subjects were asked to provide a 10-ml non-fasting venous blood sample (K3 EDTA, lavender-top tube). Colleagues were blind to all samples in the laboratory. Blood samples were centrifuged (1500–2500*g* for 15 min) within 1 h of the draw, and plasma was aliquoted into cryotubes and stored at − 20 °C. Buffy coat leukocytes were collected into another 1.5-ml Eppendorf tube, and genomic DNA was extracted using a DNeasy Blood & Tissue Kit (69506, Qiagen, Valencia CA).

### Measurement of plasma Aβ_1–42_, Aβ_1–40_, and tau

To warm up frozen human plasma samples, it was first taken to wet ice for approximately 30 min, following by keeping the plasma at room temperature for 5–10 min. For each human plasma sample, duplicated measurements of Aβ_1–42_ and Aβ_1–40_ were performed. For Aβ_1–42_ measurement, 60 μl plasma was mixed with 60 μl IMR Aβ_1–42_ reagent (MF-AB2-0060, MagQu). For Aβ_1–40_ measurement, 40 μl plasma was mixed with 80 μl IMR Aβ_1–40_ reagent (MF-AB0-0060, MagQu). Forty microliters of plasma was mixed with 80 μl IMR tau reagent (MF-TAU-0060, MagQu) for tau measurement. MF-AB0-0060, MF-AB2-0060, and MF-TAU-0060 reagents consisted of magnetic nanoparticles that were conjugated with specific antibody against Aβ1–40 protein at a.a. 1–12 (A3981, SIGMA), Aβ1–42 protein at a.a. 37–42 (ab34376, Abcam), and Tau protein at a.a. 404–441 (T9450, SIGMA), respectively. The reaction signal was recorded and analyzed with an IMR reader (XacPro-S, MagQu). Aβ_1–42_, Aβ_1–40_, and tau concentrations were obtained by converting the reaction signal via the standard curve, i.e., the relationship between Aβ_1–42_, Aβ_1–40_, or tau concentration and the reaction signal. The standard deviation (SD) of the paired measurements of Aβ_1–42_, Aβ_1–40_, and tau concentrations in plasma samples was less than 15%. The reported Aβ_1–42_, Aβ_1–40_, and tau concentrations for each plasma sample are the mean value of the duplicated measurements.

### APOE genotypes

*APOE* genotyping was performed by polymerase chain reaction (PCR) amplification of a 500-base-pair fragment of the *APOE* gene spanning amino acid positions 112 and 158, followed by direct DNA sequencing [40]. Subjects with either one or two ε4 alleles were regarded as ε4 carriers.

### Statistical methods

All statistical analysis was performed by using the Statistical Package for the Social Sciences (SPSS) software package (version 17 for Windows®, SPSS Inc., Chicago, IL, USA). Descriptive statistics for demographic, neuropsychological, and plasma biomarker data are presented as the mean ± SD. The threshold for statistical significance was *P* value < 0.05. Chi-squared tests were used to compare categorical variables between groups. General linear models with age, sex, and education as covariates were used to examine between-group differences in plasma Aβ_1–42_, Aβ_1–40_, and tau concentrations. Receiver operating characteristics (ROC) analysis and Youden’s index were performed to identify possible cut-off points, clinical sensitivities/specificities, and areas under the curve (AUCs).

## Results

All subjects were clinically suspected MCI or mild AD patients, with CDR scores of 0.5 and MMSE scores of 20–30. The demographic information for the enrolled participants is listed in Table 1.

### Amyloid PET results

According to visual rating of amyloid PET images, 22 of 52 (42.3%) subjects were amyloid PET+. Visual rating scores of 1–4 were reported for 30, three one, and 18 subjects, respectively. Rating scores of 2–4 were considered indicative of amyloid positivity. Among the 52 participants, 33 subjects are aMCI and the other 19 subjects are mild dementia. For aMCI patients, 25 (75.8%) subjects are PET− and the other 8 (24.2%) subjects are PET+. For mild dementia patients, 5 (26.3%) subjects are PET− and the other 14 (73.7%) subjects are PET+. The percentage of PET+ in dementia in this study is 73.74%, which is close to 80%.

### APOE genotypes and amyloid PET

Fourteen individuals (26.9%) were *APOE* ε4 carriers. The *APOE* ε4 carriers had significantly higher incidence of amyloid positivity than non-carriers. A total of 12 of 14 (85.7%) *APOE* ε4 carriers and 10 of 38 (26.3%) *APOE* ε4 non-carriers were amyloid PET+, as listed in Table 2.

**Table 2:** Numbers, amyloid PET positive percentage, and plasma biomarkers in *APOE* ε4 carriers and non-carriers

*APOE* genotype	ε4 carriers	ε4 non-carriers	
	ε4 carriers (ε2ε4, ε3ε4, ε4ε4)	All ε4 non-carriers (ε2ε2, ε2ε3, ε3ε3)	ε4 carriers (ε2ε2, ε2ε3)
Amyloid PET+%	85.7%	26.3%	0
Plasma Aβ_1–40_ (pg/ml)	47.1 ± 7.2	50.9 ± 7.4	50.0 ± 8.5
Plasma Aβ_1–42_ (pg/ml)	17.9 ± 2.9	16.7 ± 2.9	16.5 ± 2.7
Plasma tau (pg/ml)	24.7 ± 9.9	19.3 ± 9.5*	18.8 ± 8.2
Plasma Aβ_1–42_/Aβ_1–40_	0.396 ± 0.117	0.343 ± 0.111	0.349 ± 0.126
Plasma Aβ_1–42_xtau (pg^2^/ml^2^)	468.2 ± 262.2	348.6 ± 242.1	331.0 ± 208.6

*Abbreviations*: *Aβ* Amyloid β, *PET* positron emission tomography

*P value < 0.05: ε4 carriers vs ε4 non-carriers

#### Plasma Aβ_1–40_, Aβ_1–42_, and tau concentrations and APOE ε4

The mean plasma Aβ_1–40_ concentration of the 52 participants was 49.86 ± 7.45 pg/ml, the mean Aβ_1–42_ level was 17.04 ± 2.95 pg/ml, and the mean tau level was 20.76 ± 9.79 pg/ml.

The effect of *APOE* ε4 on plasma biomarkers was examined. The results are shown in Table 2. Demographic features including age and gender between *APOE* ε4 carriers and non-carriers were identical. *APOE* ε4 carriers showed a slightly higher plasma tau level than *APOE* ε4 non-carriers. Although not significant, *APOE* ε4 carriers showed a trend of higher levels of plasma Aβ_1–42_ and higher levels of biomarker combinations such as Aβ_1–42_/Aβ_1–40_ and Aβ_1–42_xtau than *APOE* ε4 non-carriers. In contrast, the plasma Aβ_1–40_ level was slightly lower in ε4 carriers.

### Plasma Aβ_1–40_, Aβ_1–42_, and tau and amyloid PET

**Fig. 1:**
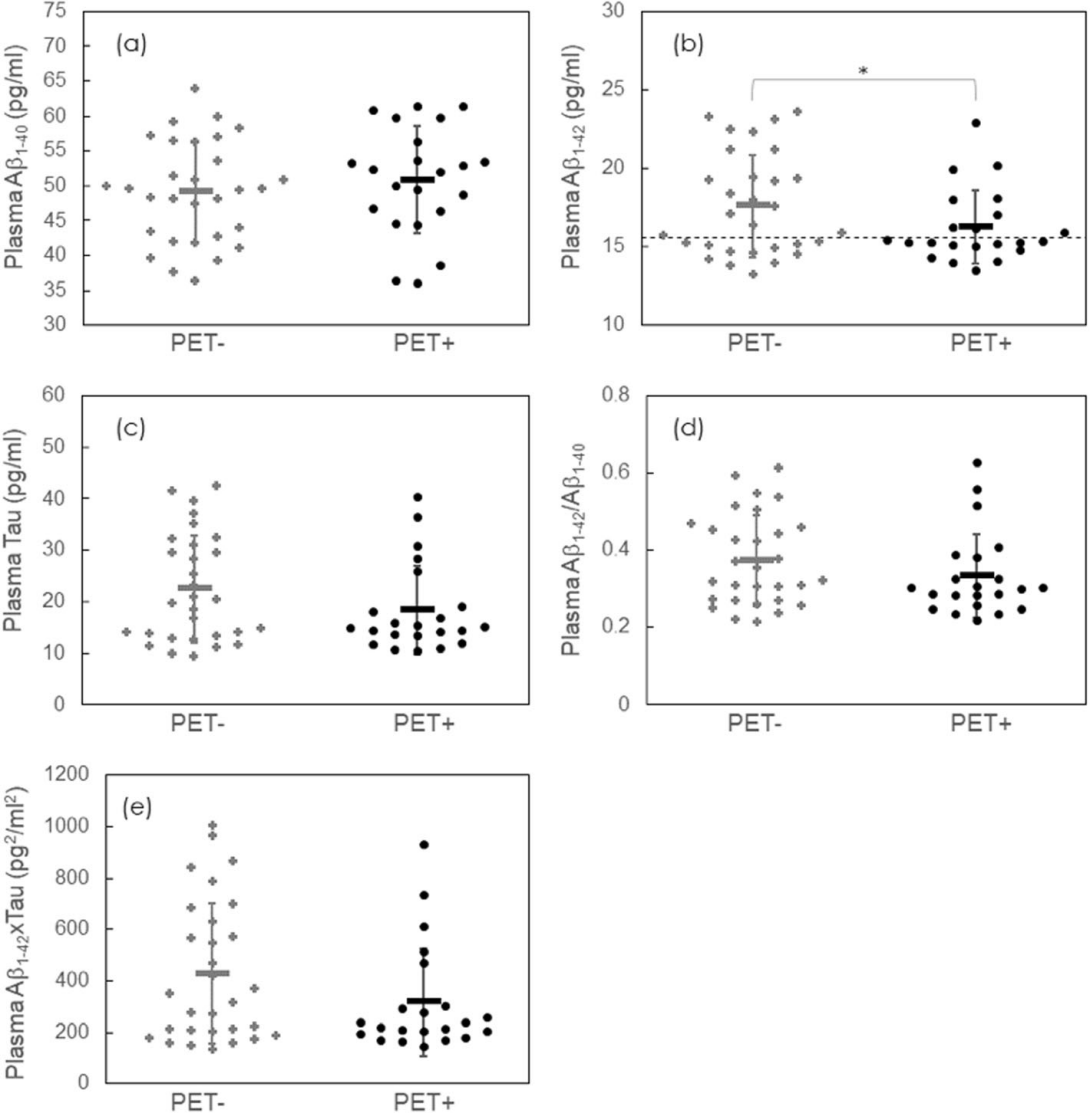
Plasma **a** Aβ_1–40_, **b** Aβ_1–42_, **c** tau, **d** Aβ_1–42_/Aβ_1–40_, and **e** Aβ_1–42_xtau for enrolled clinically suspected early stage AD subjects with negative and positive amyloid PET findings. The dashed line in **b** denotes the cut-off value, 15.58 pg/ml, to discriminate PET− from PET+ according to the ROC curve of all subjects. Abbreviations: Aβ, Amyloid β; AD, Alzheimer’s disease; PET+, amyloid positron emission tomography positivity; PET−, amyloid positron emission tomography negativity; ROC, receiver operating characteristics. *P value < 0.05

Collapsing across *APOE* genotypes, there were significant differences in both plasma Aβ_1–42_ between the amyloid PET+ and PET− groups (Fig. 1b), whereas plasma Aβ_1–40_ and tau showed no between-group difference (Fig. 1a, c). The statistical comparison in individual and combined biomarkers between amyloid PET+ and PET− are listed in Table 3. The ages and gender of PET+ and PET− are identical. *ApoE* ε4 allele frequency is much higher in PET+ (31.8%) as compared to PET− (3.33%). The amyloid PET+ group showed lower levels of plasma Aβ_1–42_ (16.3 ± 2.3 vs. 17.6 ± 3.3 pg/ml, *P* value < 0.05). However, discrimination between amyloid PET+ and PET− patients was not enhanced by using the combinations of Aβ_1–42_/Aβ_1–40_ and Aβ_1–42_xtau (Fig. 1d, e).

**Table 3:** APOE ε4 allele frequency and plasma biomarkers between amyloid positive and negative

Amyloid PET	Positive	Negative
*APOE* ε4 allele frequency	31.8%	3.33%
Plasma Aβ_1–40_ (pg/ml)	50.9 ± 7.7	49.1 ± 7.3
Plasma Aβ_1–42_ (pg/ml)	16.3 ± 2.3	17.6 ± 3.3*
Plasma Tau (pg/ml)	18.4 ± 8.5	22.5 ± 10.4
Plasma Aβ_1–42_/Aβ_1–40_	0.334 ± 0.109	0.374 ± 0.117
Plasma Aβ_1–42_xtau	316.6 ± 207.6	427.8 ± 272.1

*Abbreviations*: *Aβ* Amyloid β, *PET* positron emission tomography

*P value < 0.05

**Fig. 2:**
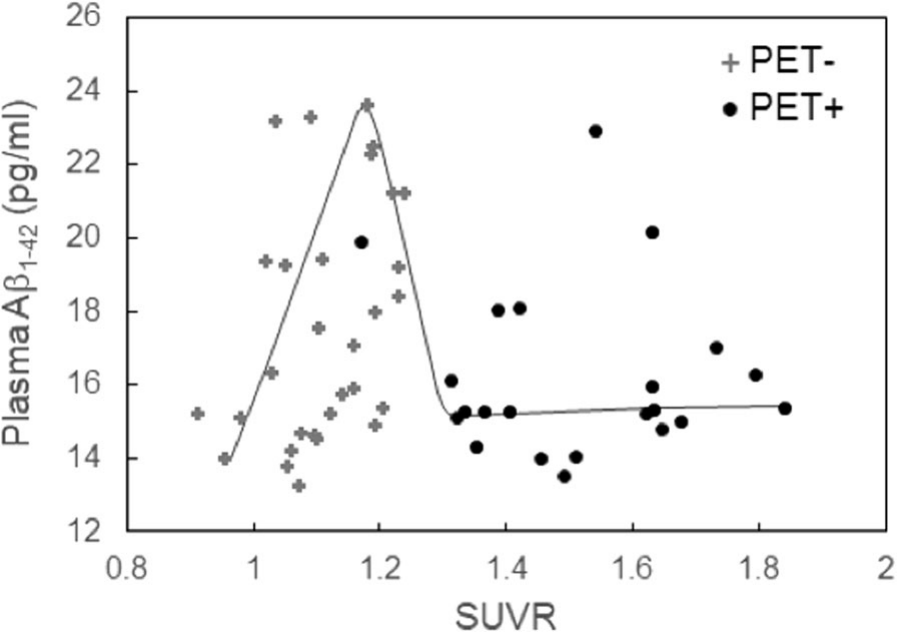
SUVR-dependent plasma Aβ_1–42_ concentrations for the 52 enrolled clinically suspected early stage AD subjects. Abbreviations: Aβ, Amyloid β; AD, Alzheimer’s disease; SUVR, standardized uptake value ratio; PET+, amyloid positron emission tomography positivity; PET−, amyloid positron emission tomography negativity

The plasma Aβ_1–42_ concentration as a function of amyloid PET SUVR is shown in Fig. 2. For PET− subjects (), the plasma Aβ_1–42_ concentration ranged from 14 to 24 pg/ml with increasing SUVR, with a mean of 17.6 pg/ml. The coefficient of correlation, *r*, between the plasma Aβ_1–42_ concentration and SUVR was 0.387, which indicates a moderate positive correlation. However, for PET+ subjects (•), the plasma Aβ_1–42_ concentration was lower than for amyloid PET− subjects, with a mean of 16.3 pg/ml (*r* = − 0.068).

### Plasma Aβ_1–42_ as a pre-screening tool for predicting amyloid PET positivity

In a ROC analysis for discriminating PET+ from PET−, the AUC was 0.611. The cut-off value for the inverse of the plasma Aβ_1–42_ level was 0.064 (pg/ml)^−1^, which equaled 15.58 pg/ml of plasma Aβ_1–42_. The corresponding sensitivity and specificity were 59.1% and 60.0%, respectively. The cut-off value is plotted with the dashed line in Fig. 1b.

**Fig. 3:**
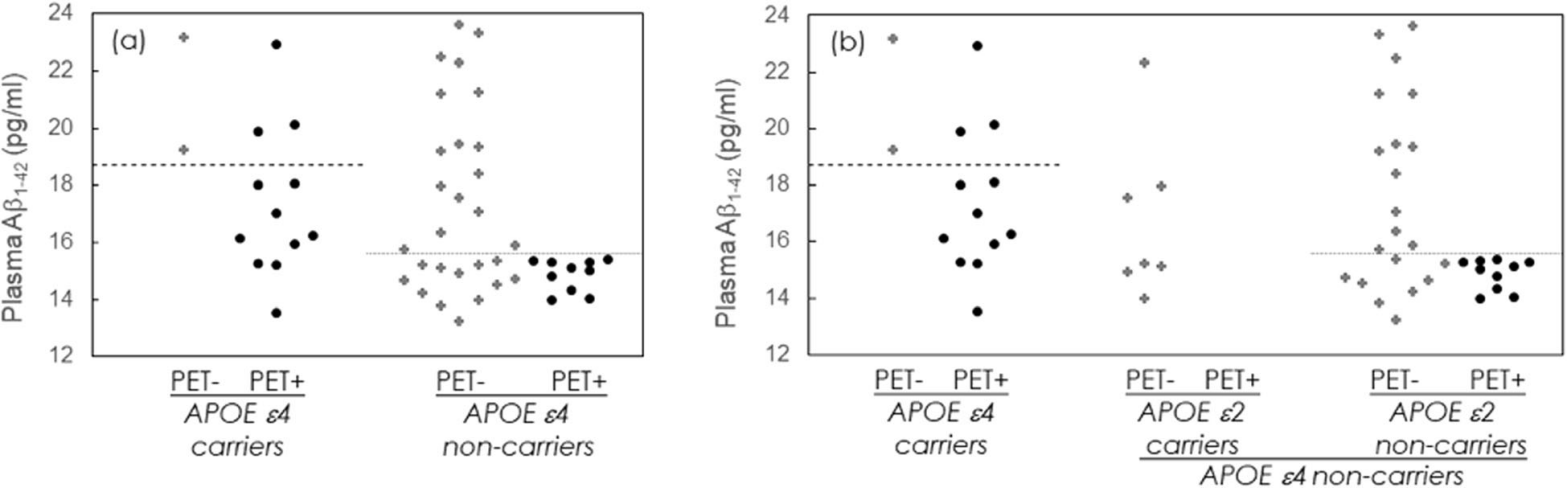
**a** Plasma Aβ_1–42_ levels in amyloid PET− and PET+ *APOE* ε4 carriers and *APOE* ε4 non-carriers. **b** Plasma Aβ_1–42_ levels in amyloid PET− and PET+ *APOE* ε4 carriers and non-carriers. *APOE* ε4 non-carriers are divided into *APOE* ε2 carriers and non-carriers. The dashed and dotted lines are the plasma Aβ_1–42_ cut-off values, 18.68 pg/ml and 15.58 pg/ml, for differentiating PET− from PET+ in *APOE* ε4 carriers and non-carriers, respectively. Abbreviations: Aβ, Amyloid β; PET+, amyloid positron emission tomography positivity; PET−, amyloid positron emission tomography negativity

Given that *APOE* ε4 status and plasma Aβ_1–42_ level enhanced the success rate in early stage AD, we further examined the contribution of combining *APOE* ε4 allele count and plasma Aβ_1–42_ to the detection of PET+ cases in those clinically suspected MCI and mild AD. The AUC was 0.826 for overall model combined with *APOE* ε4 status and plasma Aβ_1–42_ level. To determine the cut-off value of plasma Aβ_1–42_ in different conditions, we further conducted stratified ROC analysis. First, subjects were divided into two groups according to their *APOE* ε4 status, i.e., *APOE* ε4 carriers or non-carriers. Figure 3a plots the observed plasma Aβ_1–42_ levels for PET− and PET+ patients and for *APOE* ε4 carriers and non-carriers. We performed the ROC curve for discrimination of PET+ from PET− cases among clinically suspected early stage AD *APOE* ε4 carriers using (Aβ_1–42_)^−1^ as the parameter. The AUC was 0.875. The cut-off value for (Aβ_1–42_)^−1^ was found to be 0.054 (pg/ml)^−1^, which corresponds to 18.68 pg/ml of plasma Aβ_1–42._ The sensitivity and specificity were 75.0% and 100%, respectively. Remarkably, in *APOE* ε4 carriers, the success rate for detecting PET+ patients can reach 100% through enrollment of subjects with plasma Aβ_1–42_ concentrations lower than 18.68 pg/ml.

We also performed the ROC curve for differentiating PET+ from PET− in clinically suspected early stage AD *APOE* ε4 non-carriers using (Aβ_1–42_)^−1^ as a parameter. The AUC was 0.718. The cut-off value for (Aβ_1–42_)^−1^ was found to be 0.064 (pg/ml)^−1^, which corresponds to a plasma Aβ_1–42_ concentration of 15.58 pg/ml, plotted with the dotted line in Fig. 3a. The sensitivity and specificity were 100% and 57.1%, respectively. The success rate for detecting PET+ among *APOE* ε4 non-carriers was only 26.3% without assaying plasma Aβ_1–42_. Remarkably, the cut-off value in *APOE* ε4 carriers (18.68 pg/ml) was higher than that in *APOE* ε4 non-carriers (15.58 pg/ml). In *APOE* ε4 non-carriers with plasma Aβ_1–42_ lower than 15.58 pg/ml, the success rates for detecting PET+ patients were 45.5%.

After considering the effect of *APOE* ε4 status, there were still 12 PET− *APOE* ε4 non-carriers with plasma Aβ_1–42_ lower than 15.58 pg/ml (Fig. 3a). We further considered the effect of *APOE* ε2. Seven of 38 *APOE* ε4 non-carriers were *APOE* ε2 carriers. All seven *APOE* ε2 carriers among the *APOE* ε4 non-carriers were found to be PET−, as shown in Fig. 3b. Thus, we may exclude subjects with an *APOE* ε2 allele and no *APOE* ε4 allele, i.e., ε2ε2 or ε2ε3, when testing for amyloid PET+.

### Algorithm for using plasma Aβ_1–42_ as a screening tool to increase the rate of positive amyloid PET findings

**Fig. 4:**
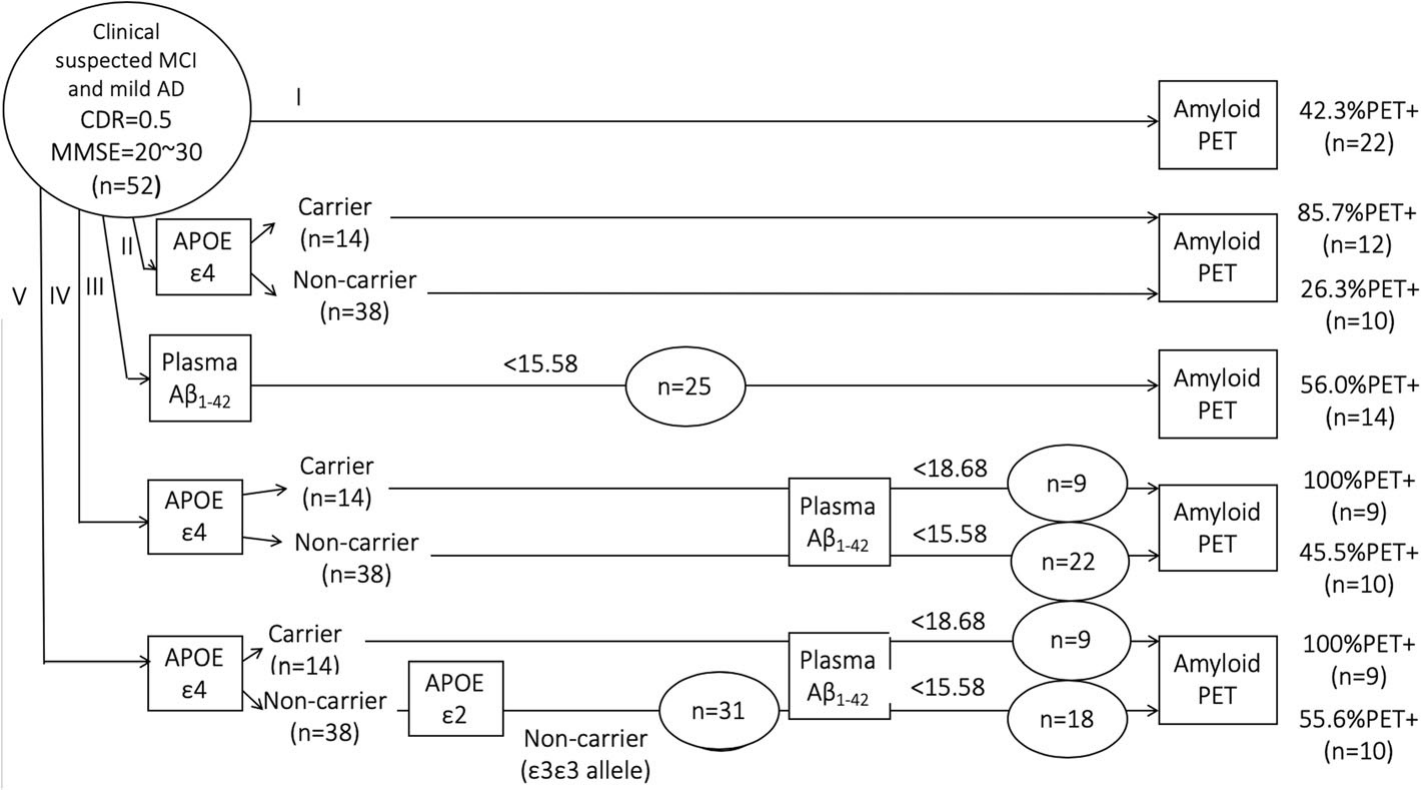
Comparison of the detection accuracy for amyloid PET+ subjects with clinically suspected mild stage AD based on genetic and plasma biomarkers. Abbreviations: Aβ, Amyloid β; AD, Alzheimer’s disease; CDR, Clinical Dementia Rating Scale; MMSE, Mini-Mental State Examination; PET+, amyloid positron emission tomography positivity; PET−, amyloid positron emission tomography negativity

The attempt to achieve the highest success rate in predicting amyloid PET+ among clinically suspected mild stage AD patients is illustrated in Fig. 4. The contributions of *APOE* genotype and plasma Aβ_1–42_ level are considered in the finalized pathway IV. With the inclusion of *APOE* ε2 status, *APOE* ε4 allele, and plasma Aβ_1–42_, the overall success rate for pathway V in predicting amyloid PET+ in clinically suspected MCI and mild dementia patients was 70.4%. The sensitivity, specificity, accuracy, positive predictive value, negative predictive value, and area under the curve for pathways II to V (utilizing various blood biomarkers for PET scan pre-screening) are presented in Table 4. The detailed information of the aMCI subgroup was presented in Additional file 1: Table S1 and Additional file 2: Figure S1.

**Table 4:** Sensitivity, specificity, positive predictive value, and negative predictive value for pathways II to V, utilizing various blood biomarkers for PET scan pre-screening

Pathway	II	III	IV	V
Biomarker	*APOE* ε4	Plasma Aβ_1–42_	*APOE* ε4 + plasma Aβ_1–42_	*APOE* ε4 + ε2 + plasma Aβ_1–42_
SS	0.595	0.591	0.864	0.864
SP	0.933	0.600	0.600	0.733
Accuracy	0.769	0.596	0.711	0.788
PPV	0.857	0.520	0.613	0.704
NPV	0.737	0.667	0.857	0.880
AUC	0.739	0.611	0.856	0.902

*Abbreviations*: *Aβ* Amyloid β, *NPV* negative predictive value, *PET* positron emission tomography, *PPV* positive predictive value, *SP* specificity, *SS* sensitivity, *AUC* area under curve

## Discussion

Published studies indicate that the occurrence of positive amyloid PET results in clinically suspected early stage AD ranges from 30 to 65%, depending on age, *APOE* genotype, and other factors [41–43]. Recent clinical trials in clinically suspected AD found a high failure rate for ascertaining amyloid PET+. An effective pre-screening tool for amyloid PET imaging is needed. In the present study, we propose an algorithm using plasma Aβ_1–42_, Aβ_1–40_, tau, and *APOE* allele status to enhance accuracy in detecting amyloid PET+ in clinically suspected early stage AD (Fig. 4). We found that the factors *APOE* genotype and plasma Aβ_1–42_ level enhance the success rate for detecting amyloid PET+ patients in clinically suspected aMCI and mild dementia.

In line with previous studies, the current study found a 42.3% occurrence of amyloid positivity in clinically suspected early stage AD patients. This result implies that the failure rate for detecting amyloid PET+ in clinically suspected early stage AD could be more than 50%. Our algorithm, combining *APOE* genotype and plasma amyloid level, increases the success rate for detecting amyloid PET+ in early-stage AD from 42.3 to 70.4%. Significantly, the success rate is especially enhanced in *APOE* ε4 non-carriers.

**Fig. 5:**
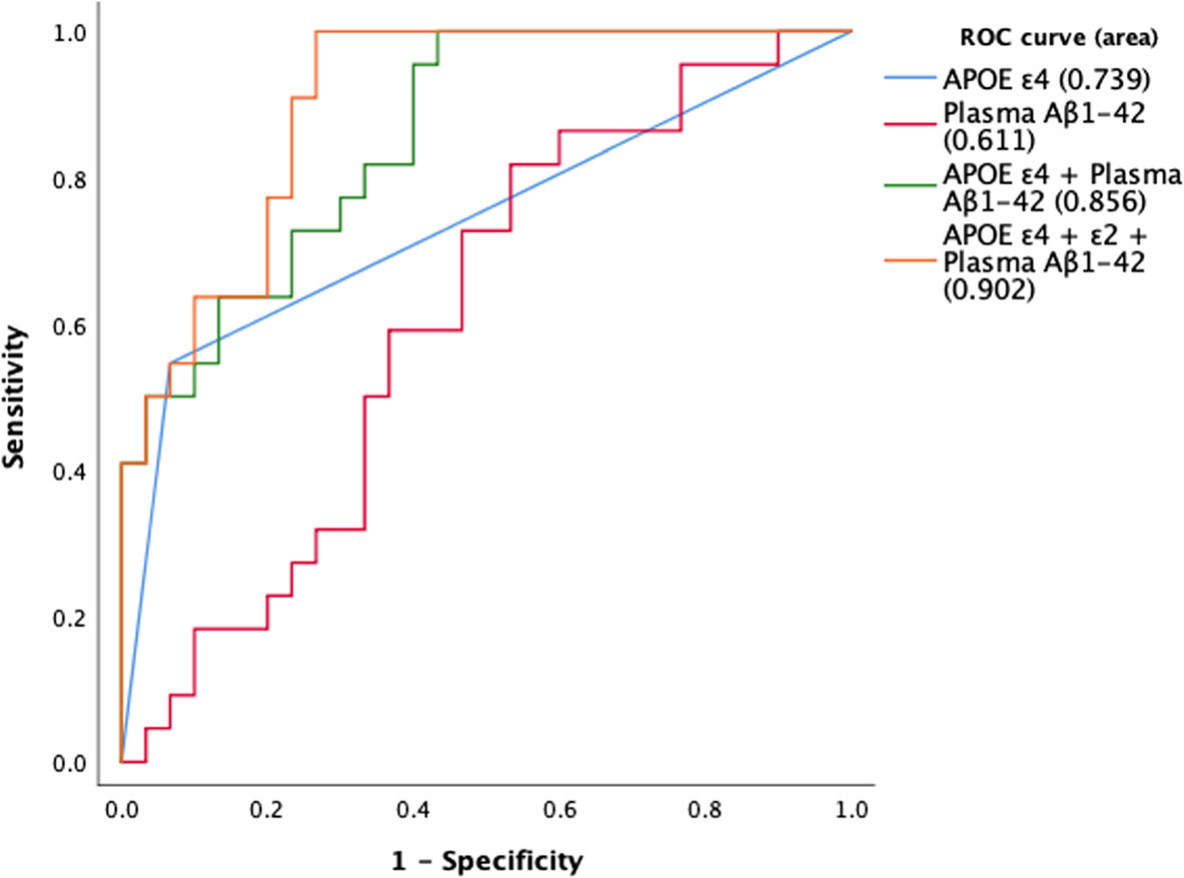
Overall receiver operating characteristics (ROC) curves for aMCI subgroups calculated in multivariate logistic models. The area under the curve (AUC) was significantly improved in combined biomarkers compared with single biomarkers (*P* = 0.014 for *APOE* ε4 alone vs *APOE* ε4 + plasma Aβ_1–42_, *P* = 0.002 for *APOE* ε4 alone vs *APOE* ε4 + *APOE* ε2 + plasma Aβ_1–42_, *P* = 0.002 for plasma Aβ_1–42_ vs *APOE* ε4 + plasma Aβ_1–42_, and *P* < 0.001 for *APOE* ε4 + *APOE* ε2 + plasma Aβ_1–42_). Abbreviations: Aβ, Amyloid β; AUC, area under the curve; ROC, receiver operating characteristics

The presence of the *APOE* ε4 allele may increase the possibility of amyloid PET+ from 42.3 to 70.4% in clinically suspected early stage AD (Fig. 4 pathway II). This result is consistent with evidence that the presence of the *APOE* ε4 allele is associated with greater amyloid deposition [41, 44, 45]. However, only approximately 30% of *APOE* ε4 non-carriers were amyloid PET+. This finding implies that *APOE* ε4 non-carriers are the population responsible for the high failure rate in detecting amyloid PET+ in clinically suspected early stage AD. However, more than 70% of subjects are *APOE* ε4 non-carriers in the general population. Even in early stage AD, more than 60% of patients are *APOE* ε4 non-carriers [30, 46, 47]. It is not possible to exclude *APOE* ε4 non-carriers in studies or trials focused on early stage AD. Other factors should be taken into account in this *APOE* ε4 non-carrier population to reduce the failure rate for detecting amyloid PET+. It has been reported that the *APOE* ε2 allele is able to prevent Aβ aggregation or plaque formation [30, 41]. Thus, the *APOE* ε2 allele should also be accounted for in predicting amyloid PET+ (Fig. 5).

Although this is a cross-sectional study, our results showed that the plasma Aβ_1–42_ concentration may increase as SUVR becomes higher in amyloid PET− patients; however, plasma Aβ_1–42_ drops steeply at the early stage of amyloid PET+ status, as indicated by the gray solid line in Fig. 2. Our findings echoed previous consensus that PET+ subjects show lower levels of plasma Aβ_1–42_ than PET− subjects [22, 28, 48, 49]. Although the longitudinal change in plasma biomarker is inconclusive [22], these results may imply that the plasma Aβ_1–42_ level becomes lower once the formation of Aβ plaques occurs in the brains of early stage AD patients. Remarkably, the correlation between CSF Aβ_1–42_ and SUVR is not a linear interrelationship and is closer to a hyperbolic model [19, 50]. In the current study, the relationship between the plasma Aβ_1–42_ concentration and SUVR may be more complicated than the hyperbolic model. Further investigations to clarify this complicated relationship should be conducted in future work. The proposed dynamic changes in plasma Aβ_1–42_ level have important implications for the selection of participants in drug trials. Amyloid-negative subjects with higher levels of Aβ_1–42_ may be not recruited for drug trials that target aggregated Aβ, but they are suitable candidates for drug trials targeting soluble Aβ in prodromal AD. A longitudinal study of the progression from PET− to PET+ status in early stage AD is needed to verify our results. Moreover, future research should investigate the biological mechanism by which changes in plasma Aβ lead to the formation of Aβ plaques in the brain.

There is limitation in this work. Given that CSF biomarkers were not used as inclusion criteria, it may happen that some of the aMCI patients do not develop AD lowering prediction rates. Another important question relates to replication/validation in an independent cohort, since the study was carried out in a small sample and individual results showed high variability.

## Conclusion

According to the results of the current study, based on a limited sample of clinically suspected aMCI and mild AD subjects, combining *APOE* genotypes and plasma Aβ_1–42_ increases the accuracy for detecting amyloid PET+ in early stage AD from 42.3 to 70.4%. Plasma Aβ_1–42_ cut-off values for discriminating PET+ from PET− patients are proposed for *APOE* ε4 carriers and non-carriers. The information reported in the current study may help pharmaceutical companies to effectively enroll clinically suspected early stage AD subjects with Aβ plaques in the brain. Future longitudinal studies should be conducted to clarify the biological mechanism for the revolution of the plasma Aβ_1–42_ level during the progression from amyloid PET− to PET+ stage.

## Supplementary information

**Additional file 1 : Table S1.** Sensitivity, specificity, positive predictive value, and negative predictive value for the pathways II to V, utilizing various blood biomarkers for PET scan pre-screening in aMCI populations.

**Additional file 2 : Figure S1.** Overall receiver operating characteristic (ROC) curves of each diagnostic pathways calculated in multivariate logistic models. The area under the curve (AUC) was significantly improved in combined biomarkers compared with *APOE* ε4 alone (*P* = 0.027 for *APOE* ε4 + Plasma Aβ_1–42_, *P* = 0.005 for *APOE* ε4 + *APOE* ε2 + Plasma Aβ_1–42_). There was still a trend of better AUC compared with Plasma Aβ_1–42_ alone (*P* = 0.339 for *APOE* ε4 + Plasma Aβ_1–42_, *P* = 0.130 for *APOE* ε4 + *APOE* ε2 + Plasma Aβ_1–42_). Abbreviations: Aβ, Amyloid β; aMCI, amnestic mild cognitive impairment; AUC, area under the curve, AUC; ROC, receiver operating characteristics

## Data Availability

The datasets generated during the current study are available from the corresponding author on reasonable request.

## Abbreviations

Aβ: Amyloid β
AD: Alzheimer’s disease
ADNI: Alzheimer’s Disease Neuroimaging Initiative
aMCI: Amnestic mild cognitive impairment
*APOE*: Apolipoprotein E
AUCs: Areas under the curve
CDR: Clinical Dementia Rating Scale
CGMH: Chang Gung Memorial Hospital
CSF: Cerebrospinal fluid
3-D OSEM: Three-dimensional ordered subset expectation maximization
IMR: Immunomagnetic reduction
LM: Logical memory
MMSE: Mini-Mental State Examination
MNI: Montreal Neurological Institute
MR: Magnetic resonance
NC: Normal cognition
NIA-AA: National Institute on Aging and the Alzheimer’s Association
PET: Positron emission tomography
PET+: Positron emission tomography positive
PET-: Positron emission tomography negative
PCR: Polymerase chain reaction
ROC: Receiver operating characteristics
SD: Standard deviation
SPSS: Statistical Package for the Social Sciences
SUVR: Standardized uptake value ratio
T-ADNI: Alzheimer’s Disease Neuroimaging Initiative in Taiwan
Taipei VGH: Taipei Veterans General Hospital
VOI: Volumes of interest
WMS-III: Wechsler Memory Scale-III
