# Supplementary material for: Plasma amyloid assay as a pre-screening tool for amyloid positron emission tomography imaging in early stage Alzheimer’s disease
Source: Alzheimers Res Ther. 2019 Dec 27;11:111. doi: 10.1186/s13195-019-0566-0 (PMC6933740; doi:10.1186/s13195-019-0566-0)
Supplement: Supplementary file 1 — Additional file 1 : Table S1. Sensitivity, specificity, positive predictive value, and negative predictive value for the pathways II to V, utilizing various blood biomarkers for PET scan pre-screening in aMCI populations. [file 13195_2019_566_MOESM1_ESM.docx]

Table S1: Sensitivity, specificity, positive predictive value, and negative predictive value for the pathways II to V, utilizing various blood biomarkers for PET scan pre-screening in aMCI populations.

| Pathway | II | III | IV | V |
| --- | --- | --- | --- | --- |
| Biomarker | *APOE* ε4 | Plasma Aβ_1-42_ | *APOE* ε4 +  Plasma Aβ_1-42_ | *APOE* ε4 + ε2 +  Plasma Aβ_1-42_ |
| SS | 0.375 | 0.75 | 1 | 1 |
| SP | 0.88 | 0.56 | 0.48 | 0.56 |
| Accuracy | 0.757 | 0.606 | 0.606 | 0.666 |
| PPV | 0.50 | 0.352 | 0.380 | 0.421 |
| NPV | 0.814 | 0.875 | 1 | 1 |
| AUC | 0.648 | 0.695 | 0.800 | 0.850 |

Abbreviation: Aβ, Amyloid β; NPV, negative predictive value; PET, positron emission tomography; PPV, positive predictive value; SP, specificity; SS, sensitivity; AUC, area under curve
