# Supplementary figures and images for: Plasma amyloid assay as a pre-screening tool for amyloid positron emission tomography imaging in early stage Alzheimer’s disease
Source: Alzheimers Res Ther. 2019 Dec 27;11:111. doi: 10.1186/s13195-019-0566-0 (PMC6933740; doi:10.1186/s13195-019-0566-0)

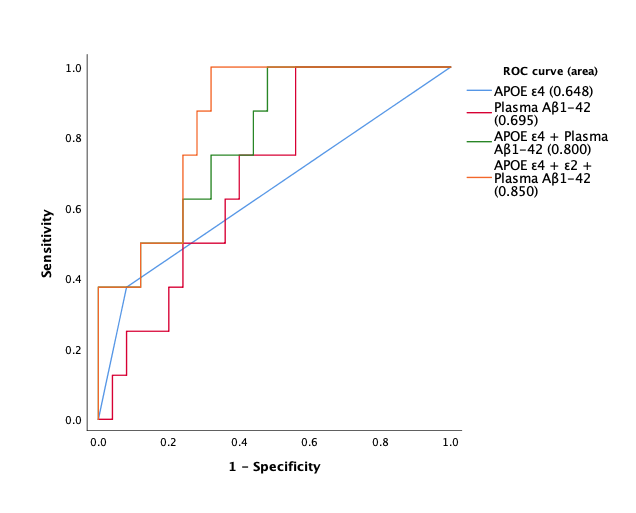

Supplement: Supplementary file 2 — Additional file 2 : Figure S1. Overall receiver operating characteristic (ROC) curves of each diagnostic pathways calculated in multivariate logistic models. The area under the curve (AUC) was significantly improved in combined biomarkers compared with APOE ε4 alone (P = 0.027 for APOE ε4 + Plasma Aβ1–42, P = 0.005 for APOE ε4 + APOE ε2 + Plasma Aβ1–42). There was still a trend of better AUC compared with Plasma Aβ1–42 alone (P = 0.339 for APOE ε4 + Plasma Aβ1–42, P = 0.130 for APOE ε4 + APOE ε2 + Plasma Aβ1–42). Abbreviations: Aβ, Amyloid β; aMCI, amnestic mild cognitive impairment; AUC, area under the curve, AUC; ROC, receiver operating characteristics [file 13195_2019_566_MOESM2_ESM.tif]
